# HIF-1*α* determines the metastatic potential of gastric cancer cells

**DOI:** 10.1038/sj.bjc.6604919

**Published:** 2009-02-17

**Authors:** N Rohwer, S Lobitz, K Daskalow, T Jöns, M Vieth, P M Schlag, W Kemmner, B Wiedenmann, T Cramer, M Höcker

**Affiliations:** 1 Department of Hepatology and Gastroenterology, Charité, Campus Virchow-Klinikum, Berlin 13353, Germany; 2 Laboratory for Angiogenesis and Tumour Metastasis, Charité, Campus Virchow-Klinikum, Berlin 13353, Germany; 3 Freie Universität Berlin, Fachbereich Biologie, Chemie, Pharmazie, Berlin 14195, Germany; 4 Department of Pediatric Oncology and Hematology, Charité, Campus Virchow-Klinikum, Berlin 13353, Germany; 5 Institute of Integrative Neuroanatomy, Center for Anatomy, Charité, Campus Mitte, Berlin 10115, Germany; 6 Institute of Pathology, Klinikum Bayreuth, Bayreuth 95445, Germany; 7 Surgical Oncology Research Group, Max Delbrück Center for Molecular Medicine, Berlin 13125, Germany

**Keywords:** HIF-1*α*, hypoxia, metastasis, gastric cancer

## Abstract

Gastric adenocarcinoma is characterised by rapid emergence of systemic metastases, resulting in poor prognosis due to vanished curative treatment options. Better understanding of the molecular basis of gastric cancer spread is needed to design innovative treatments. The transcription factor HIF-1*α* (hypoxia-inducible factor 1*α*) is frequently overexpressed in human gastric cancer, and inhibition of HIF-1*α* has proven antitumour efficacy in rodent models, whereas the relevance of HIF-1*α* for the metastatic phenotype of gastric adenocarcinoma remains elusive. Therefore, we have conducted a comprehensive analysis of the role of HIF-1*α* for pivotal metastasis-associated processes of human gastric cancer. Immunhistochemistry for HIF-1*α* showed specific staining at the invading tumour edge in 90% of human gastric cancer samples, whereas normal gastric tissue was negative and only a minority of early gastric cancers (T1 tumours) showed specific staining. Hypoxia-inducible factor 1*α*-deficient cells showed a significant reduction of migratory, invasive and adhesive properties *in vitro*. Furthermore, the HIF-1*α*-inhibitor 2-methoxy-estradiol significantly reduced metastatic properties of gastric cancer cells. The accentuated expression at the invading edge together with the *in vitro* requirement of HIF-1*α* for migration, invasion and adherence argues for a pivotal role of HIF-1*α* in local invasion and, ultimately, systemic tumour spread. These results warrant the exploration of HIF-1*α*-inhibiting substances in clinical treatment studies of advanced gastric cancer.

Gastric adenocarcinoma is the second leading cause of cancer-related deaths worldwide with an annual incidence of 80–90/100 000 cases in Japan and 5–15/100 000 in Europe (Parkin, 2001). Surgical resection remains the only curative treatment option; however, most patients emerge at advanced clinical stages and typically show lymphatic tumour dissemination at the time of diagnosis (Hohenberger and Gretschel, 2003). Here, surgical resection under curative intent is no longer achievable leading to a poor prognosis with 5-year survival rates of less than 30%. Therefore, a better understanding of the molecular mechanisms governing local invasion and systemic spread of gastric cancer is needed to design and evaluate new therapeutic strategies for this lethal disease.

Lack of oxygen (hypoxia) is a hallmark of solid tumour formation and constitutes an independent prognostic factor in a diverse range of malignant tumours (Harris, 2002; Vaupel *et al*, 2004). Hypoxia is associated with local invasion, metastatic spread, resistance to radio- as well as chemotherapy and, ultimately, poor patient prognosis in many human carcinomas (Vaupel *et al*, 2004). The transcription factor HIF-1 (hypoxia-inducible factor 1) constitutes the principal mediator of cellular adaptation to hypoxia (Poellinger and Johnson, 2004). Hypoxia-inducible factor 1 is a heterodimeric protein consisting of a ubiquitously expressed *β*-subunit (also known as ARNT (aryl hydrocarbon receptor nuclear translocator)) and a hypoxia-inducible *α*-subunit (Semenza, 2003). Hypoxia-inducible factor 1*α* expression has been shown in a vast array of human carcinomas and their metastases by means of immunohistochemistry (Zhong *et al*, 1999). For breast, colorectal, invasive cervical and squamous cell oesophageal cancer a positive correlation between tumoural HIF-1*α* expression and prognosis has been shown (Birner *et al*, 2000; Bos *et al*, 2003; Kurokawa *et al*, 2003; Kuwai *et al*, 2003).

Several immunhistochemical studies showed that HIF-1*α* is overexpressed in gastrointestinal stromal tumours of the stomach (Takahashi *et al*, 2003; Chen *et al*, 2005) and gastric adenocarcinomas but absent in normal gastric mucosa (Mizokami *et al*, 2006; Sumiyoshi *et al*, 2006; Urano *et al*, 2006; Griffiths *et al*, 2007). Although two studies have assessed HIF-1*α* as a prognostic marker in gastrointestinal stromal tumours of the stomach (Takahashi *et al*, 2003; Chen *et al*, 2005), conflicting data concerning patient prognosis for gastric adenocarcinoma exist (Mizokami *et al*, 2006; Sumiyoshi *et al*, 2006; Urano *et al*, 2006; Cabuk *et al*, 2007; Griffiths *et al*, 2007). Inhibition of HIF-1*α* by means of RNA interference or chemical compounds has proven antitumoural activity in two murine gastric cancer models. Treatment of subcutaneous xenografts of the human gastric cancer cell line NCI-H87 in nude mice with an HIF-1*α*-inhibiting compound resulted in smaller and less vascularised tumours (Yeo *et al*, 2003). In addition, when the human gastric cancer cell line TMK-1 stably expressed a dominant negative form of HIF-1*α*, tumours grew slower, showed smaller overall vessel area and hampered vessel maturation when implanted orthotopically in nude mice (Stoeltzing *et al*, 2004). However, besides the well characterised effects of HIF-1*α* on angiogenesis and vessel maturation, a molecular mechanism for the proposed inhibitory action of blocking HIF-1*α* on gastric cancer is lacking and the precise relevance of HIF-1*α* for the causal pathogenesis of gastric cancer is not well defined. To explore the functional role of HIF-1*α* for the metastatic capacity of human gastric cancer cells, we designed a lentiviral-mediated RNA-interference system to knockdown HIF-1*α in vitro*. In a complementary pharmacological approach, we used the HIF-1*α*-inhibitor 2-methoxy-estradiol (2ME2) to investigate its effect on the migratory and adhesive ability of gastric cancer cells. Our results showed that, while HIF-1*α* was dispensable for cellular proliferation, functional and pharmacological inactivation of the factor lead to a significant reduction of migratory, invasive and adhesive features of human gastric cancer cells *in vitro*. Hence, we characterised for the first time the functional importance of HIF-1*α* for central cell biological properties of metastatic human gastric cancer cells.

## Materials and methods

### Study population and tissues

A tissue microarray comprising tumours from patients (*n*=52) who underwent curative (*R*_0_) gastrectomy between 1995 and 2003 at the Division of Surgery and Surgical Oncology (Robert Rössle Hospital, Charité Campus Buch, Berlin) was used. Written informed consent for experimental immunohistochemistry was obtained from all patients before analyses. The study was approved by the Ethics Committee of the Charité, Berlin. Patients’ age ranged from 34 to 85 years (mean 62.7±11.2 years), 41% were female (*n*=17) and 59% male (*n*=24). Four patients were lost during follow-up and thus censored at the time of last contact (mean follow-up time 37.7±27.3 months). In one patient, the cause of death was unknown and the death of another six patients was not related to gastric cancer. Thirty-nine patients showed disease relapse and 34 patients had died at the end of the study due to gastric cancer. Staging and diagnosis of gastric carcinoma was assessed according to the WHO classification (WHO Classification. Tumours of the Digestive System, 2000) and the TNM classification set out by the International Union against cancer (Wittekind *et al*, 2003). Pre-neoplastic tissues, such as intestinal metaplasia and dysplasia, have not been subjected to immunohistochemistry. All gastric cancers in this study were adenocarcinomas of the gastric body, signet ring cell carcinomas and adenocarcinomas from the gastroesophageal junction were excluded from the analysis. In addition, we included 40 specimens from patients with early gastric cancers (EGCs), histologically defined as T1 tumours confined to the mucosal or submucosal layer but not beyond. No clinicopathological data from these patients were available for this study.

### Cell culture and chemicals

The human gastric cancer cell lines AGS (CRL-1739, ATCC, Rockville, MD, USA) and MKN28 (JCRB Cell Bank, Tokyo, Japan) and the human embryonic kidney cell line 293T (CRL-11268, ATCC) were grown in monolayer cultures in recommended medium. Human umbilical vein endothelial cells were obtained from PromoCell (Heidelberg, Germany) and cultured in endothelial cell growth medium supplemented with Supplement Mix and 2% fetal calf serum (PromoCell). Desferrioxamine mesylate salt (DFO) and 2ME2 were purchased from Sigma-Aldrich (Deisenhofen, Germany).

### Immunohistochemistry and evaluation of immunostaining

Immunohistochemical detection of HIF-1*α* on human paraffin sections was done as described in detail before (Pfander *et al*, 2005). Tissue samples were independently scored by an expert pathologist (MV), who was blinded for clinical data. Staining results for HIF-1*α* was classified by calculating the percentage of epithelial cells showing specific immunoreactivity: negative (0–10% positive nuclei), weak (10–30% positive nuclei), moderate (30–60% positive nuclei), strong (>60% positive nuclei). Only samples showing moderate or strong immunoreactivity were considered positive. Correlation of immunohistochemical results with clinicopathological parameters was performed for an exploratory purpose.

### Plasmid construction and generation of cell lines stably expressing siRNAs

Short hairpin RNA sequences against human HIF-1*α* and scrambled (SCR) control oligonucleotides (TIB MOLBIOL, Berlin, Germany) were published elsewhere (Sowter *et al*, 2003; Mizukami *et al*, 2004). Oligonucleotides were inserted into *BsrG*I and *Xba*I restriction sites of the lentiviral bicistronic vector pPR1, which allows for coexpression of GFP (Leurs *et al*, 2003). Recombinant lentiviruses were produced by transient transfection of pPR-HIF-1*α* or pPR-scr with packaging vectors in 293T cells using the calcium-phosphate method (Szulc *et al*, 2006). Vector titres were determined by transducing 293T cells with serial dilutions of concentrated lentivirus, and GFP was used to quantitate the transduced cell fraction by flow cytometry 60 h after transduction. Gastric cancer cell lines stably expressing siRNAs were generated by double transduction with lentiviruses at a multiplicity of infection of 10 for 24 h. Transduction efficiency of target cells was determined by flow cytometry analysis of GFP using a FACSCalibur (Becton Dickinson, Heidelberg, Germany).

### Western blot analysis

Nuclear protein extracts were prepared as described in detail before (Cramer *et al*, 2008), then resolved by electrophoresis on an 8% sodium dodecyl sulphate-polyacrylamide gel, and transferred onto a nitrocellulose membrane (Amersham Biosciences, Freiburg, Germany). The blots were probed with monospecific HIF-1*α* (AB1536; R&D Systems, Minneapolis, MN, USA) and YY1 (sc-281; Santa Cruz Biotechnology, Santa Cruz, CA, USA) antibodies. Immunreactive proteins were visualised using the Western Lightning Chemiluminescence Reagent Plus (Perkin Elmer Life Sciences, Boston, MA, USA).

### Quantitative real-time PCR analysis

For real-time PCR analysis, total cellular RNA was extracted with Trizol reagent (Invitrogen, Rockville, MD, USA). First strand cDNA was synthesised with an oligo (dT) primer and a SuperScript First Strand Synthesis System (Invitrogen). For PCR reactions, TaqMan PCR Universal Mastermix (for actin and phosphoglycerate kinase) or SYBR GREEN PCR Master Mix (for HIF-1*α*-KD; Applied Biosystems, Darmstadt, Germany) were used. Quantitative real-time PCR analysis was performed as described in detail before (Cramer *et al*, 2003). Knockdown frequency of HIF-1*α* was determined with the following primers: HIF-1*α*-KD forward 5′-CCGCTGGAGACACAATCATA-3′, HIF-1*α*-KD reverse 5′-CTTCCTCAAGTTGCTGGTCA-3′.

### Transient transfection and reporter luciferase assay

Cell lines AGS and MKN28 were co-transfected with 100 ng of pHRE-Luc and 30 ng of phRL-null (Promega, Mannheim, Germany) using Effectene Transfection Reagent (Qiagen, Hilden, Germany) according to the manufacturer's protocol. Measurement of luciferase activity with the Dual Luciferase Reporter Assay System (Promega) was performed as described before (Cramer *et al*, 2008).

### Cell proliferation assay

Cells (3 × 10^4^) were seeded in triplicate into 24-well plates, and 18 h later cells were placed under normoxic or hypoxic culture. Cells were trypsinised and counted every 2 days using a hemacytometer. Medium was not changed for the duration of the experiment.

### Migration and invasion assay

Cells (1 × 10^5^) in serum-free DMEM or RPMI 1640 medium were seeded in duplicate into uncoated Costar transwell inserts (8 *μ*m pore size; Corning Costar Co., Bodenheim, Germany) for migration assays or Matrigel-coated transwell inserts (8 *μ*m pore size, BD Biosciences, Heidelberg, Germany) for invasion assays and incubated for 24 h at 37°C in 5% CO_2_ atmosphere under normoxia (20% O_2_) or hypoxia (1% O_2_). Random as well as directed migration and invasion assays were performed and analysed as described in detail before (Cramer *et al*, 2003).

### Adhesion assay

For adhesion assay, human umbilical vein endothelial cells were grown to confluence on 24-well plates. AGS cells were suspended in the amount of 8 × 10^5^ cells ml^−1^ in serum-free medium and labelled with 10 *μ*M BCECF/AM (Calbiochem, Darmstadt, Germany) by 30-min incubation at 37°C. Labelled AGS (2 × 10^5^ per well) cells were then incubated with the human umbilical vein endothelial cell monolayer for 30 min at 37°C. Cultures were washed twice with PBS to remove non-adherent AGS cells. Adherence was quantified by measuring fluorescence of attached AGS cells using excitation of 485 nm and emission of 535 nm.

### Statistical analysis

Statistical analysis was carried out using Prism 4.0 software (GraphPad Software Inc., San Diego, CA, USA). Statistical significance was determined by unpaired two-tailed *t*-test (*P*<0.05). Patient data were analysed using the SPSS software (SPSS Inc., Chicago, Il, USA). Survival was determined from the date of surgery to the time of event (diagnosis of recurrence or death) using the Kaplan–Meier method. Relationships between positivity for HIF-1*α* and clinicopathological features were evaluated using Spearman's rank correlation coefficient (ordinally scaled parameters) or Fisher's exact probability test (dichotome parameters). Statistical significance of differences in cumulative survival curves was evaluated using the log-rank test.

## Results

### Expression pattern of HIF-1*α* in human gastric cancer and non-transformed gastric tissues

**Figure 1:**
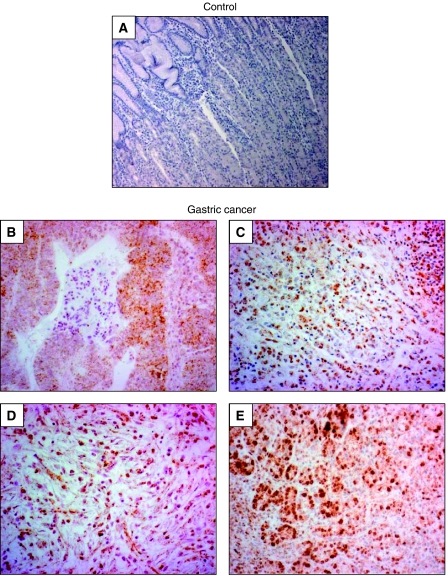
Expression pattern of HIF-1*α* in human gastric cancer tissues. Paraffin sections were pretreated as described in Materials and Methods, and HIF-1*α* was visualised by means of immunohistochemistry. (**A**) Negative control staining. (**B**–**E**) Expression of HIF-1*α* in established human gastric cancers, showing that the vast majority of tumour cells were positively stained for HIF-1*α* over nuclei. Magnification × 100 (**A** and **B**) and × 200 (**C**–**E**).

Immunohistochemistry with a monospecific, polyclonal HIF-1*α* antibody showed no specific staining in normal gastric mucosa (Supplementary Figure 1A). Furthermore, analysis of 40 cases of EGC defined as all T1 gastric carcinomas that are confined to the mucosal or submucosal layer but not beyond failed to detect HIF-1*α* protein in tumour cells (Supplementary Figure 1B and C). However, infiltrating inflammatory cells were frequently positive for HIF-1*α* (not shown). In sharp contrast, 90% of analysed gastric cancer samples showed positivity for HIF-1*α* specifically over the nuclei of neoplastic epithelial cells (Figure 1B–E). Interestingly, no difference in HIF-1*α* staining intensity was noted when well-differentiated cancers were compared with poorly differentiated ones. Hypoxia-inducible factor 1*α* positive neoplastic epithelial cells did not show a preferential distribution with respect to tissue architecture and were scattered unevenly throughout the tumour. The staining pattern therefore did not resemble a hypoxia-induced HIF-1*α* expression, but rather the HIF-1*α* stabilisation was observed to result from oncogene gain of function and tumour suppressor gene loss of function, respectively. Notably, comparable with the EGC samples, tumour-infiltrating inflammatory cells steadily showed a specific nuclear HIF-1*α* staining (not shown). Statistical analysis of patient data with the HIF-1*α* status failed to detect a significant association of HIF-1*α* staining with venous invasion, lymphatic invasion, lymph node metastasis or tumour stage (Table 1). However, due to the small number of patients who completed the follow-up (*n*=41), this investigation can solely describe a tendency of robust expression of HIF-1*α* in gastric cancer cells. A valid statistical analysis could only be performed with the help of larger patient cohorts.

### Expression and activity of HIF-1*α* in gastric cancer cells

**Figure 2:**
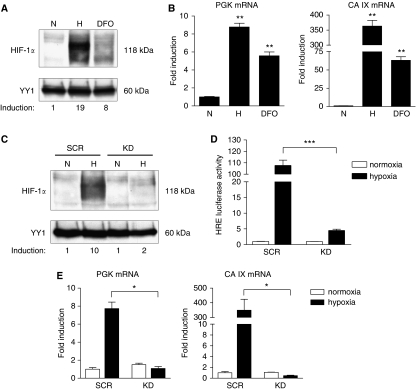
Expression and inhibition of HIF-1*α* in the human gastric cancer cell line AGS. Cells were cultured under normoxia (N) or hypoxia (H) or treated with 100 *μ*M DFO. (**A**) HIF-1*α* protein levels were analysed in nuclear extracts by western blot analysis, with YY1 serving as nuclear loading control. Induction of HIF-1*α* protein was quantified by densitometry. Hypoxia-inducible factor 1 protein was found to be expressed in AGS cells under both hypoxia and DFO treatment. (**B**) Transcription of the HIF-1 target genes 3-phosphoglycerate kinase (PGK) and carbonic anhydrase IX (CA IX) was analysed by quantitative real-time PCR. Hypoxic culture and treatment with DFO resulted in induction of PGK (^**^P⩽0.0086) and CA IX (^**^P⩽0.0060) mRNAs. Values were normalised to that of *β*-actin, and relative expression was compared with the same cell line in hypoxia or DFO treatment *vs* normoxia. Values are means±s.e.m. (**C**) Western blot analysis of nuclear extracts from knockdown (KD) and control (SCR) AGS cells under normoxia (N) or hypoxia (H) for 16 h. AGS KD cells were unable to express HIF-1*α* protein under hypoxic culture. Differences in hypoxia-induced nuclear HIF-1*α* protein levels were quantified by densitometry. (**D**) Confirmation of loss of HIF-1*α* function by HRE-luc reporter assay. AGS KD and SCR cells were co-transfected with an HRE-luc reporter and phRL-null *Renilla* as an internal control and incubated under either normoxia or hypoxia for 24 h. Inhibition of HIF-1*α* resulted in a significant decrease of HRE-luc reporter activity under hypoxic conditions (^***^P<0.0001). Luciferase activity, normalised to *Renilla* luciferase activity, was expressed relative to that of transfected control cells (SCR) under normoxia, set at 1.0. Results shown are representative of three independent experiments, and values represent the mean±s.e.m. of triplicate determinations. (**E**) Expression of HIF-1 target genes PGK and CA IX was measured relative to *β*-actin by quantitative real-time PCR. Inhibition of HIF-1*α* protein by RNAi resulted in decreased transcription of HIF-1 target genes PGK (^*^P=0.0125) and CA IX (^*^P=0.0421) in AGS KD cells. Transcription levels were expressed relative to that of control cells (SCR) under normoxia, set at 1.0. Values represent the means±s.e.m.

The role of HIF-1*α* for the malignant progression of gastric cancer was studied *in vitro* by using the two human gastric cancer cell lines AGS and MKN28. As shown by western blot analysis, in both cell lines HIF-1*α* protein was strongly induced by hypoxia (1% O_2_) and the hypoxia-mimicking agent DFO (Figure 2A and Supplementary Figure 2A). Under ambient oxygen conditions (20%), HIF-1*α* protein expression was not detectable in AGS and MKN28 cells. Next, the effect of hypoxia and DFO on mRNA expression of two known HIF-1 target genes, phosphoglycerate kinase (PGK) and carbonic anhydrase IX (CA IX), was determined by quantitative real-time PCR. As expected, hypoxia and DFO significantly induced mRNA expression of both target genes in AGS and MKN28 cells, whereas hypoxic induction of these genes was higher than induction by DFO (Figure 2B and Supplementary Figure 2B).

### Inhibition of HIF-1*α* by RNA interference in gastric cancer cells

To determine the effects of HIF-1*α* inactivation on gastric cancer progression, we developed a lentivirus-based system for stably expressing siRNA against HIF-1*α*. Human gastric cancer cells AGS and MKN28 were transduced with lentiviral vectors containing the H1 promoter-driven human HIF-1*α* siRNA (knockdown, ‘KD’) or unspecific control siRNA (scrambled, ‘SCR’) (Leurs *et al*, 2003). Efficiency of HIF-1*α* knockdown was identified by locus-specific real-time PCR analysis and showed in AGS KD cells a mean knockdown of 85.2±6.3% and in MKN28 KD of 97.2±2.1%. Furthermore, western blot analysis showed a near complete failure of AGS KD and MKN28 KD cells to induce HIF-1*α* protein under hypoxic conditions (Figure 2C and Supplementary Figure 2C). To further characterise the functional inhibition of HIF-1*α*, we determined mRNA expression of the HIF-1 target genes PGK and CA IX as outlined above. As shown in Figure 2E, hypoxia induced the expression of these genes in both control cell lines, whereas hypoxic induction of these genes in AGS KD and MKN28 KD cells was markedly reduced (Supplementary Figure 2E). To confirm that not only HIF-1*α* protein and HIF-1 target gene expression but also HIF-1*α* activity was reduced in KD cells, an HRE-luciferase reporter assay was performed. Hypoxic stimulation resulted in a significant decrease of HRE-luc reporter activity in AGS KD and MKN28 KD cells compared with their respective control cells (Figure 2D and Supplementary Figure 2D). Taken together, these results show that our lentivirus-based siRNA system lead to a strong and stable inactivation of HIF-1*α* in a different panel of human gastric cancer cell lines.

### Effects of HIF-1*α* inhibition on proliferation of gastric cancer cells *in vitro*

**Figure 3:**
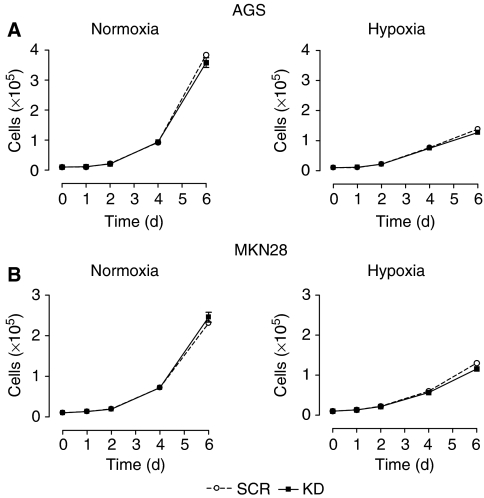
Effects of HIF-1*α* inhibition on growth of AGS and MKN28 cells *in vitro*. Anchorage-dependent proliferation of AGS (**A**) and MKN28 (**B**) knockdown (KD) and control (SCR) cells under normoxia (left panels) or hypoxia (right panels). Cells were counted every other day from day 2 to 6 using a hemacytometer. Gastric cancer KD cells grew at approximately the same rate as SCR cells under both normoxia and hypoxia. Results shown are representative of three independent experiments and values represent the mean±s.e.m. of duplicate determinations.

Given the widespread acceptance of HIF-1*α* as a positive regulator of cellular proliferation (Dang *et al*, 2006), we characterised the consequences of HIF-1*α* inhibition on gastric cancer growth. As can be seen in Figure 3, loss of HIF-1*α* did not interfere with proliferation of AGS and MKN28 cells under anchorage-dependent conditions neither in normoxic nor hypoxic conditions. However, during hypoxic culture, gastric cancer cells were not able to sustain exponential growth, and both KD and control cells had significant decreases in cell numbers compared with cell growth under ambient oxygen conditions (Figure 3A and B; right panels). These results suggest that HIF-1*α* is not essential for cell proliferation of these two gastric cancer cell lines under adherent conditions.

### Inhibition of the metastatic cascade *in vitro* through functional inactivation of HIF-1*α*

**Figure 4:**
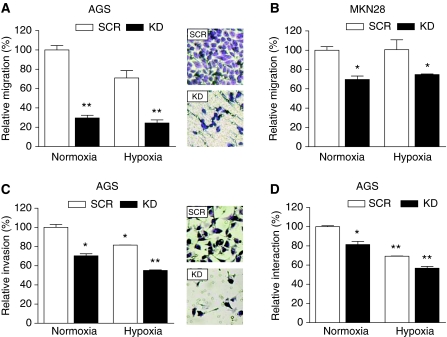
Effects of HIF-1*α* inactivation on migration, invasion and adhesion of AGS and MKN28 cells. Migration of AGS (**A**) and MKN28 (**B**) knockdown (KD) and control (SCR) cells was evaluated in 24-well Transwell chambers (8 *μ*m pore size) under normoxia or hypoxia for 24 h. Directed migration of KD cells was significantly impaired compared with SCR cells under both normoxia and hypoxia (^**^P⩽0.0056; ^*^P⩽0.0281). The number of migrated cells on the bottom side of the filter was determined and normalised to the number of migrated SCR cells under normoxic conditions. Data represent mean±s.e.m. of a representative out of three experiments, each performed in duplicate. Photos show the bottom of representative migration filters. (**C**) For invasion assay, AGS KD and SCR cells were seeded into Matrigel-coated transwell inserts (8 *μ*m pore size) and incubated under normoxia or hypoxia for 24 h. Hypoxia decreased the invasion of both AGS KD and SCR cells. Inactivation of HIF-1*α* reduced directed invasion of AGS cells significantly under normoxia and hypoxia (^*^P⩽0.0266; ^**^P=0.0048). The number of invading cells was normalised to the number of invading SCR cells under normoxic conditions. Bars show means±s.e.m. of a representative experiment out of two experiments, each performed in duplicate. Photos show the bottom of representative invasion filters. (**D**) For adhesion assay, BCECF/AM labelled AGS KD and SCR cells were added to an HUVEC monolayer and allowed to adhere for 30 min under normoxia or hypoxia. Adhesion of AGS cells to HUVEC endothelial cells was reduced under normoxic and hypoxic conditions by loss of HIF-1*α* (^*^P=0.0327; ^**^P⩽0.0025). Adhesion was expressed relative to that of control cells (SCR) under normoxic conditions. Shown are mean±s.e.m. of three independent experiments, each performed in triplicate.

Next, we determined the impact of HIF-1*α* inactivation on functional aspects of malignant cell behaviour, including migration, invasion and interaction with endothelial cells. To determine if HIF-1*α* is involved in gastric cancer cell motility, we compared the migratory and invasive capacity of HIF-1*α* KD and control cells. Directed migration of AGS cells was significantly reduced by the loss of HIF-1*α* under both normoxic and hypoxic conditions to 30 and 24%, respectively (Figure 4A). Similarly, inactivation of HIF-1*α* in MKN28 cells reduced directed migration to 70% under both normoxic and hypoxic conditions (Figure 4B). In good agreement with our observations on migration, loss of HIF-1*α* in AGS cells reduced directed invasion to 70% under normoxia and to 55% under hypoxic conditions when compared with control cells under normoxia (Figure 4C). Generally, directed migration and invasion of AGS cells was decreased under hypoxic conditions when compared with normoxia (Figure 4A and C).

### Inhibition of HIF-1*α* reduces adhesion to the endothelium *in vitro*

Lymphatic and blood vessels are the major pathways for cancer cell dissemination. Interactions between circulating intravascular cancer cells and endothelial cells have a significant influence on the fate of metastatic cells and on the outcome of the metastatic process. To evaluate the direct impact of HIF-1*α* on interaction between malignant cells and the endothelium, cell adhesion assays were performed. As shown in Figure 4D, inhibition of HIF-1*α* significantly reduced adhesion of AGS cells to endothelial cell monolayers to 81% under normoxia and to 57% in hypoxic culture compared with relative adhesion of control cells under normoxia. Furthermore, the adhesion of both AGS KD and AGS SCR cells was lower under hypoxic conditions than under normoxia (Figure 4D).

### 2ME2 inhibits HIF-1*α* and reduces the metastatic capacity of gastric cancer cells

**Figure 5:**
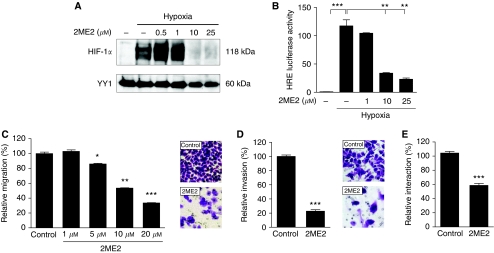
Effects of 2ME2 on HIF-1*α* protein and transcriptional activity and on metastatic properties of AGS cells. (**A**) AGS cells were treated for 24 h with vehicle or increasing concentrations of 2ME2, and inhibition of HIF-1*α* was analysed by western blot analysis. Treatment with 2ME2 led to a dose-dependent reduction of HIF-1*α* protein. (**B**) HRE-luc reporter assay. AGS cells transfected with an HRE-luc reporter, and phRL-null *Renilla* were treated with vehicle or increasing concentrations of 2ME2 under either normoxia or hypoxia for 24 h. Exposure to 2ME2 resulted in a significant decrease of HRE-luc reporter activity under hypoxic conditions (^***^P=0.0004; ^**^P⩽0.0089). Luciferase activity, normalised to *Renilla* luciferase activity, was expressed relative to that of transfected control cells under normoxia, set at 1.0. Results shown are representative of three independent experiments, and values represent the mean±s.e.m. of triplicate determinations. (**C**) Migration of AGS cells was evaluated in 24-well transwell chambers (8 *μ*m pore size) after treatment with either vehicle or increasing concentrations of 2ME2 under normoxia for 24 h. Directed migration of 2ME2-treated cells was significantly impaired compared with vehicle-treated cells (^*^P=0.0246; ^**^P=0.002; ^***^P=0.001). The number of migrated cells on the bottom side of the filter was determined and normalised to the number of vehicle-treated cells. Results shown are representative of three independent experiments, and values represent the mean±s.e.m. of duplicate determinations. Photos show the bottom of representative migration filters. (**D**) For invasion assay, AGS cells were seeded into Matrigel-coated transwell inserts (8 *μ*m pore size) after pretreatment with either vehicle or 10 *μ*M 2ME2 for 24 h. Invasion of 2ME2-treated cells was significantly reduced compared with vehicle-treated cells (^***^P<0.0001). Bars show means±s.e.m. of a representative experiment out of two experiments, each performed in duplicate. Photos show the bottom of representative invasion filters. (**E**) For adhesion assay, AGS cells were pretreated with either vehicle or 10 *μ*M 2ME2 for 24 h, labelled with BCECF/AM and allowed to adhere to HUVEC endothelial cells for 30 min. Treatment with 2ME2 significantly reduced adhesion of AGS cells to an HUVEC monolayer (^***^P=0.0002). Shown are means±s.e.m. of two independent experiments, each performed in triplicate.

2-methoxy-estradiol, a naturally occurring metabolite of estradiol, is an orally active small molecule with antitumour and antiangiogenic activity currently in phase I/II clinical trials (Schumacher and Neuhaus, 2006). It has been shown that 2ME2 downregulates HIF-1 protein at the posttranscriptional level and inhibits its transcriptional activity (Mabjeesh *et al*, 2003). We first examined the effects of 2ME2 treatment on HIF-1*α* protein in the gastric cancer cell line AGS. As shown in Figure 5A, exposure of AGS cells to 2ME2 reduced the levels of nuclear HIF-1*α* protein under hypoxia in a dose-dependent manner. To further investigate the effects of 2ME2 treatment on HIF-1 transcriptional activity, we performed an HRE-luciferase reporter assay as described before. Consistent with the reduced levels of HIF-1*α* nuclear protein, treatment with 2ME2 resulted in a significant and dose-dependent decrease of hypoxia-induced transcriptional activity of HIF-1*α* (Figure 5B). Owing to the antimetastatic acitivity of 2ME2, we investigated whether treatment with 2ME2 attenuates metastatic properties of the gastric cancer cell line AGS. Therefore, AGS cells were pretreated with increasing concentrations of 2ME2 for 24 h and migration, invasion and adhesion assays were performed. As shown in Figure 5C, reduced cell migration was observed in AGS cells treated with 5 *μ*M 2ME2 but a more significant reduction was found after treating the cells with 10 and 20 *μ*M 2ME2 compared with the control. Similarly, treatment of AGS cells with 10 *μ*M 2ME2 led to a significant suppression of directed invasion to 17% compared with invasion of vehicle-treated cells (Figure 5D). Finally, inhibition of HIF-1*α* by means of 2ME2 significantly reduced adhesion of AGS cells to endothelial cell monolayers to 59% compared with relative adhesion of vehicle-treated cells (Figure 5E).

## Discussion

Here, we describe for the first time that HIF-1*α* constitutes a central metastasis-supporting factor in human gastric adenocarcinoma. We provide experimental evidence for reduced metastatic properties of gastric cancer cells through functional inactivation of HIF-1*α*, even under conditions of ambient oxygen. Our characterisation of the temporospatial expression patterns of HIF-1*α* protein during the pathogenesis of human gastric adenocarcinoma are in line with earlier studies showing HIF-1*α* protein expression in the majority of human gastric cancer samples (Urano *et al*, 2006; Cabuk *et al*, 2007; Griffiths *et al*, 2007). In contrast, two Japanese studies reported lower percentages of HIF-1*α*-positive tumour cells in human gastric adenocarcinoma samples (Mizokami *et al*, 2006; Sumiyoshi *et al*, 2006). Absolute patient numbers and tissue types were comparable in these studies, ruling out an impact on HIF-1*α* distribution. However, significant differences in staining procedures and scoring protocols were noted and constitute the most likely explanation for the reported differences in the relative number of HIF-1*α*-positive tumour cells. Our analysis of EGC samples detected specific nuclear expression of HIF-1*α* in only 15% of the examined cases. Although this result is in line with one previous study showing specific HIF-1*α* staining in 29% of T1 tumours (Sumiyoshi *et al*, 2006), two other articles reported strikingly different results (HIF-1*α* positivity in 68 and 100% of T1 tumours) (Urano *et al*, 2006; Cabuk *et al*, 2007). Given these sharp contrasts, a definite agreement on the expression pattern of HIF-1*α* in T1 gastric adenocarcinomas cannot be reached at this point and should be clarified by future work. We noted robust nuclear expression of HIF-1*α* at the invading tumour edge, confirming previously published results (Sumiyoshi *et al*, 2006). Hence, tissue-infiltrating gastric tumour cells seemed particularly reliant on HIF-1*α*. These results lead us to speculate that the role of HIF-1*α* in the pathogenesis of human gastric cancer might be most pronounced in advanced stages of the disease. Therefore, we performed an array of *in vitro* experiments aimed at defining the importance of HIF-1*α* for local invasion and metastatic spread of gastric cancer cells.

These analyses showed robust induction of HIF-1*α* protein expression by hypoxia and the hypoxia-mimicking agent DFO, whereas normoxic expression of HIF-1*α* could not be observed in AGS and MKN28 cells. Hypoxic induction of HIF-1*α* in AGS cells has been described by others before and is confirmed by our data (Huang *et al*, 2005; Cai *et al*, 2006). However, MKN28 cells have not been analysed for the expression profile of HIF-1*α* thus far. Induction of HIF-1*α* protein and target gene expression by culture under hypoxic conditions has been described for a wide variety of human cancer cell lines, for example, colon, pancreas, breast and prostate cancer (Zhong *et al*, 1998; Ravi *et al*, 2000; Akakura *et al*, 2001; Tacchini *et al*, 2004). Two basic expression patterns of HIF-1*α* in cancer cell lines are known: inducible and constitutive expression (Akakura *et al*, 2001). The latter being explained by the observation that loss of tumour-suppressor genes, for example, *vhl*, or activation of oncogenes such as *ras* or *src* can directly activate HIF-1*α* (Jiang *et al*, 1997; Maxwell *et al*, 1999; Blancher *et al*, 2001). Our results show that in both gastric cancer cell lines investigated, HIF-1*α* protein is not detectable under normoxic conditions by means of western blot. However, despite the lack of detectable protein expression, HIF-1*α* can still be functional under normoxic conditions. This has been observed by us and others before and is most likely explained by technical detection limitations due to the extremely short half-life of HIF-1*α* under normoxic conditions (Semenza, 2002; Cramer *et al*, 2003).

Proliferation of AGS and MKN28 cells showed similar results for HIF-1*α*-deficient and control cells. Interestingly, this result was noted both for normoxic and hypoxic culture conditions, arguing for a neglectable role of HIF-1*α* for both normoxic and hypoxic proliferation of these cell types. These results stand in contrast to the analysis of immortalised murine embryonic fibroblasts, where a genetic inactivation of HIF-1*α* resulted in a substantial growth defect under hypoxia (Seagroves *et al*, 2001). Furthermore, murine mammary tumour cells from mice with Cre-loxP-mediated inactivation of HIF-1*α* failed to reach the logarithmic growth phase under low oxygen conditions (Liao *et al*, 2007). In line with these observations, human colon cancer cell lines HCT116 and RKO showed a growth defect under both normoxic and hypoxic conditions when HIF-1*α* was inactivated (Dang *et al*, 2006). Hence, our analysis for the first time describes a HIF-1*α*-independent proliferation pattern of malignant cells with respect to oxygen concentration. Therefore, HIF-1*α* seems negligible for the growth of AGS and MKN28 cells under anchorage-dependent conditions.

As outlined above, most patients present with advanced disease due to either local invasion of neighbouring organs or the occurrence of distant metastases. Once the primary tumour has spread systemically, no curative treatment options remain and the treatment goal is purely palliative. Therefore, continued research efforts to better understand the molecular mechanisms of systemic tumour spread are urgently needed. The classical metastatic cascade encompasses entry of blood vessels by tumour cells, their survival in the vascular network, arrest in distant organs, extravasation and ultimately growth into metastatic foci (Bogenrieder and Herlyn, 2003; Steeg, 2006). Our functional analysis of the role of HIF-1*α* in the pathogenesis of human gastric cancer comprised central characteristics of metastatic cells, namely migration, invasion and adhesion to endothelial cells (Steeg, 2006). Functional inactivation of HIF-1*α* resulted in a significantly reduced ability of AGS and MKN28 gastric cancer cells to migrate and to invade an artificial basal membrane under normoxic as well as hypoxic conditions. Furthermore, HIF-1*α*-competent cells showed a significantly better adherence to endothelial cells without reference to the oxygen concentration. Reduced migratory capacitiy of HIF-1*α*-deficient cells has been shown for a wide variety of primary and transformed cells, for example, neutrophils, macrophages, glioma and small cell lung cancer cells (Cramer *et al*, 2003; Liu *et al*, 2006; Zagzag *et al*, 2006). In the case of primary murine phagocytes, a highly reduced content of intracellular ATP has been shown to be the underlying molecular mechanism, most likely due to the pivotal role of HIF-1*α* for glycolysis (Seagroves *et al*, 2001; Cramer *et al*, 2003). However, in our experiments we could neither show lowered ATP levels in HIF-1*α*-deficient gastric cancer cells nor did addition of excess free ATP rescue the motility of HIF-1*α*-deficient cells (data not shown). Various research groups have reported a permissive role of HIF-1*α* for the invasive capacity of transformed and non-transformed cell types (Cramer *et al*, 2003; Miyoshi *et al*, 2006; Zagzag *et al*, 2006). Here, HIF-1*α*-controlled expression of matrix metalloproteinase 2, cathepsin D and urokinase plasminogen activator receptor have been implicated among others as key molecular players (Semenza, 2003). The precise mechanism(s) of the reduced invasive capacity of AGS and MKN28 gastric cancer cells without functional HIF-1*α* has not been determined here and remains a topic for future research.

Research on the oncogenic role of HIF-1*α* has gained significant attention in recent years. Hypoxia-inducible factor 1*α* expression is associated with limited survival and poor treatment outcome in breast, brain, colon and lung cancer. Functional inhibition of HIF-1*α* by means of dominant negative expression constructs or stable siRNA transfection led to significant reduction of neoplastic growth in a broad array of rodent tumour models. 2-methoxy-estradiol has been shown by independent international research groups to potently inhibit HIF-1*α in vitro* and *in vivo*. These preclinical studies led to the realisation of clinical phase I and II trials aimed at testing the safety and efficacy of 2ME2 as a novel anticancer drug. Our results show a potent inhibiting effect of 2ME2 on protein expression and activity of HIF-1*α* in gastric cancer cells. These results are well in line with other studies showing an anti-HIF-1*α* effect of 2ME2, for example, on breast, colon and pancreatic cancer cells. Furthermore, we were able to characterise a substantial inhibiting effect of 2ME2 on the migratory, invasive and adhesive properties of gastric cancer cells. These results confirm our experiments with the RNAi-mediated HIF-1*α* inhibition and therefore firmly establish a functional role of HIF-1*α* for migration and invasion of gastric cancer cells. Against this background and given the widespread expression of HIF-1*α* in human gastric cancer samples, it seems justified to include 2ME2 (or other equally effective HIF-1*α*-inhibiting agents) in study protocols against gastric cancer.

Taken together, we performed a detailed analysis of the role of HIF-1*α* for human gastric cancer *in vitro* and *in vivo*. Hypoxia-inducible factor 1*α* was expressed in about 90% of late gastric cancer samples, whereas the factor could not be identified in EGCs. Functional inhibition of HIF-1*α* by means of lentiviral-mediated siRNA delivery resulted in suppression of a diverse range of metastasis-associated pathways *in vitro*. Hence, our data argue for a pivotal role of HIF-1*α* in the pathogenesis of later stages of human gastric cancer and its systemic spread. These results warrant the design and execution of clinical studies to test the efficacy of inhibitors of HIF-1*α* in the treatment of patients with gastric cancer.

## Figures and Tables

**Table 1:** Relationship between HIF-1*α* protein expression in tumour tissues from tissue microarrays and clinicopathological parameters

	**No.**	**HIF-1*α* (%)**	***P*-value**
*Venous invasion*			
Absent	29 (71%)	95.00	0.065
Present	12 (29%)	99.58	
			
*Lymphatic invasion*			
Absent	24 (59%)	96.88	0.582
Present	17 (41%)	95.59	
			
*Lymph node metastases (pN0-3)*			
N0	11 (27%)	95.91	0.820
N1	20 (49%)	95.75	
N2	6 (15%)	98.33	
N3	4 (10%)	97.50	
			
*Tumour infiltration (pT)*			
T1	7 (17%)	98.57	0.378
T2	20 (49%)	95.75	
T3	11 (27%)	95.00	
T4	3 (7%)	100.00	
