# Supplementary figures and images for: HIF-1α determines the metastatic potential of gastric cancer cells
Source: Br J Cancer. 2009 Feb 17;100(5):772–81. doi: 10.1038/sj.bjc.6604919 (PMC2653758; doi:10.1038/sj.bjc.6604919)

**NORMAL STOMACH**

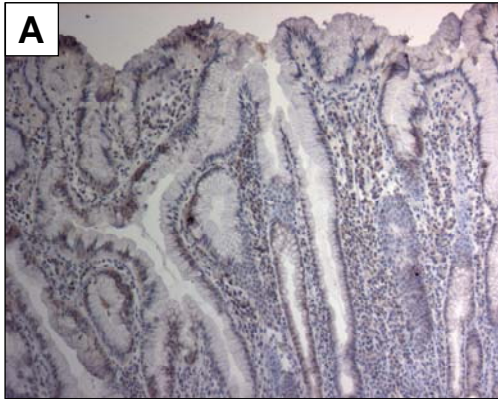

**EARLY GASTRIC CANCER**

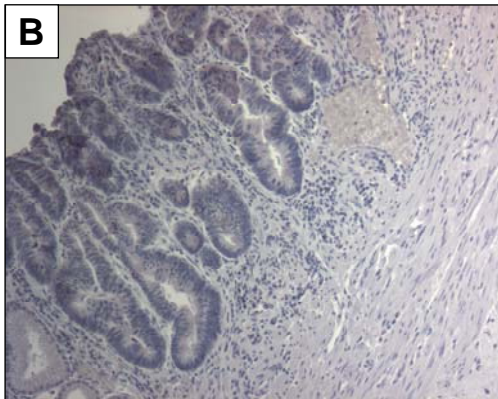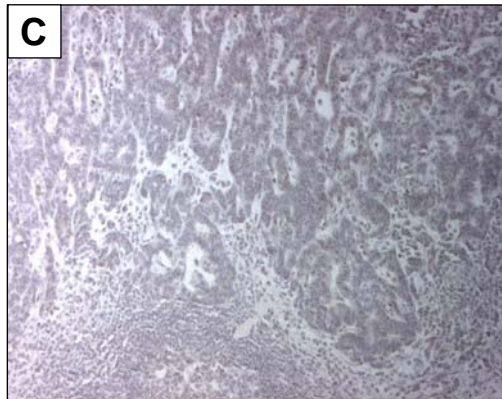

**A**

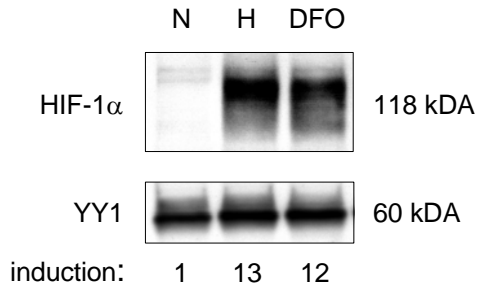

**B**

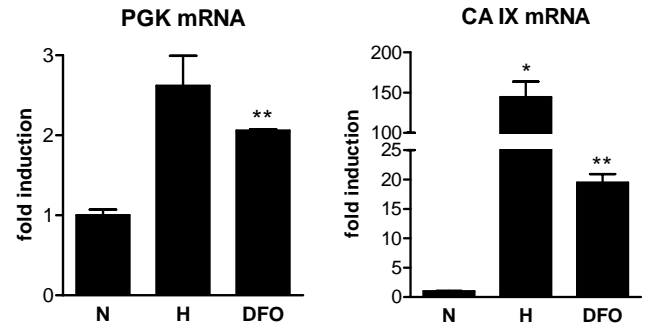

**C**

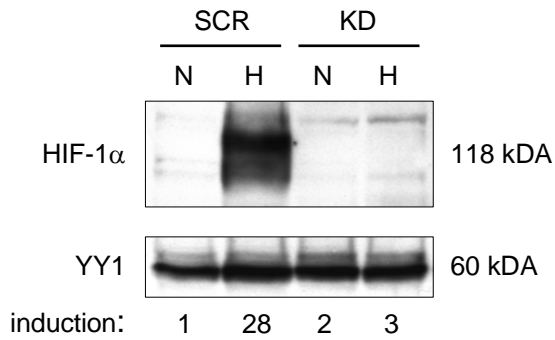

**D**

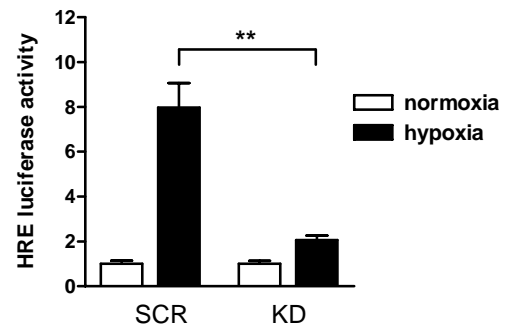

**E**

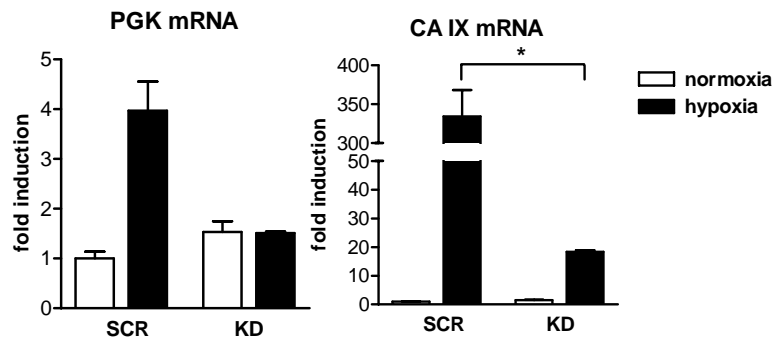

Supplement: Supplementary Figure 1 and 2 [file 6604919x1.pdf]
