# Supplementary material for: HIF-1α determines the metastatic potential of gastric cancer cells
Source: Br J Cancer. 2009 Feb 17;100(5):772–81. doi: 10.1038/sj.bjc.6604919 (PMC2653758; doi:10.1038/sj.bjc.6604919)
Supplement: Supplementary Figure Legends [file 6604919x2.doc]

**Figure Legends**

**Supplementary Figure 1** Expression pattern of HIF‑1α in human normal mucosa and early gastric cancer tissues (EGC). Paraffin sections were pretreated as described in materials and methods and HIF‑1α was visualized by means of immunohistochemistry. Epithelial cells in normal mucosa (**A**) did not display specific signal for HIF‑1α, neither did neoplastic cells in EGC (**B** and **C**). Magnification x100.

**Supplementary Figure 2** Expression and inhibition of HIF‑1α in MKN28 gastric cancer cells. Cells were cultured under normoxia (N), hypoxia (H) or treated with 100 µM DFO. (**A**) Nuclear extracts were assayed by western blot analysis with a HIF‑1α specific antibody. HIF‑1α protein was found to be expressed in MKN28 cells under both hypoxia and DFO treatment. (**B**) Transcription of the HIF‑1 target genes 3-phosphoglycerate kinase (PGK) and carbonic anhydrase IX (CA IX) was analyzed by quantitative real-time PCR. Hypoxic culture and treatment with DFO resulted in induction of PGK (**, *P* = 0.0046) and CA IX (*, *P* = 0.0173; **, *P* = 0.059) mRNAs. Values are mean ± SEM. (**C**) Inactivation of HIF‑1α by RNA interference was analyzed by western blot analysis for HIF‑1α. MKN28 KD cells were unable to express HIF‑1α protein under hypoxic culture. (**D**) Loss of HIF‑1α function was confirmed by HRE-luc reporter assay. Shown is a representative (mean ± SEM) of three independent experiments, each performed in triplicates. RNA interference against HIF‑1α resulted in a significant decrease of HRE‑luc reporter activity under hypoxic conditions (**, *P* = 0.0059). (**E**) Expression of HIF‑1 target genes PGK and CA IX was measured relative to β-actin by quantitative real-time PCR. Inhibition of HIF‑1α protein by RNAi resulted in decreased transcription of HIF‑1 target genes PGK and CA IX (*, *P* = 0.0115) in MKN28 KD cells. Values represent the means ± SEM.
